# Surfactant-Free Stabilization of Aqueous Graphene Dispersions Using Starch as a Dispersing Agent

**DOI:** 10.1021/acsomega.1c00699

**Published:** 2021-04-28

**Authors:** Wei Zhao, Abhilash Sugunan, Thomas Gillgren, Johan A. Larsson, Zhi-Bin Zhang, Shi-Li Zhang, Niklas Nordgren, Jens Sommertune, Anwar Ahniyaz

**Affiliations:** † RISE Research Institutes of Sweden, Stockholm SE-114 86, Sweden; ‡ BillerudKorsnäs AB, Frövi SE-718 80, Sweden; § Division of Solid State Electronics, Department of Electrical Engineering, Uppsala University, Uppsala SE-751 03, Sweden

## Abstract

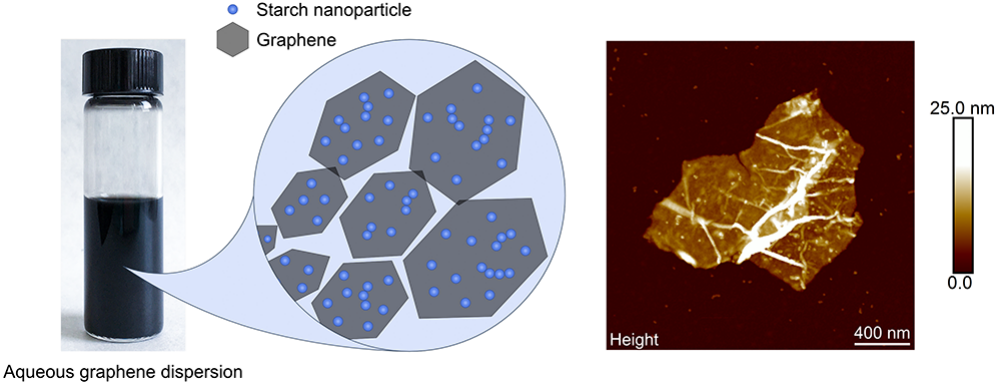

Attention to graphene dispersions in water with the aid of natural polymers is increasing with improved awareness of sustainability. However, the function of biopolymers that can act as dispersing agents in graphene dispersions is not well understood. In particular, the use of starch to disperse pristine graphene materials deserves further investigation. Here, we report the processing conditions of aqueous graphene dispersions using unmodified starch. We have found that the graphene content of the starch–graphene dispersion is dependent on the starch fraction. The starch–graphene sheets are few-layer graphene with a lateral size of 3.2 μm. Furthermore, topographical images of these starch–graphene sheets confirm the adsorption of starch nanoparticles with a height around 5 nm on the graphene surface. The adsorbed starch nanoparticles are ascribed to extend the storage time of the starch–graphene dispersion up to 1 month compared to spontaneous aggregation in a nonstabilized graphene dispersion without starch. Moreover, the ability to retain water by starch is reduced in the presence of graphene, likely due to environmental changes in the hydroxyl groups responsible for starch–water interactions. These findings demonstrate that starch can disperse graphene with a low oxygen content in water. The aqueous starch–graphene dispersion provides tremendous opportunities for environmental-friendly packaging applications.

## 1 Introduction

Graphene is a two-dimensional (2D) network of sp^2^-hybridized carbon atoms in a hexagonal configuration that gives rise to an exceptional combination of mechanical, thermal, and electronic properties. Owing to these properties, graphene is extensively studied for various applications in a wide range of fields, such as composites, energy storage, data communication, electronics, sensors, and biomedical technologies.^1^ Since the first isolation of graphene,^2^ there has been a growing interest in understanding its surface chemistry^3,4^ and developing more sustainable dispersing systems.^5,6^ For instance, dispersing graphene in water instead of organic solvents can offer safer handling and greater biocompatibility, while diminishing adverse impact on human health and the environment. However, graphene is not dispersible in water due to a large mismatch between the low surface energy of graphene and the high surface tension of water.^7^ Moreover, attractive interactions (van der Waals force, π–π stacking, and hydrophobic interactions) that can exist between adjacent graphene sheets lead to restacking and eventually aggregation. The restacking of graphene sheets can be prevented by introducing electrostatic or steric repulsion between the sheets using amphiphilic dispersing agents.^8−10^ Great efforts have been devoted to the stabilization of graphene by noncovalent modifications using surfactants, which is a relatively simple process from an industrial perspective. However, the use of surfactant systems introduces challenges such as foaming and surface migration in polymer matrices that can complicate most graphene applications.^11,12^ Furthermore, the increasing occurrence of commercial synthetic surfactants and their degraded products in the environment is receiving more attention due to their adverse effects on the ecosystem.^13,14^ Attractive alternatives to surfactants for the preparation of aqueous graphene dispersions are biopolymers. Among biopolymers, starch is one of the most abundant polymers in nature with attractive rheological, adhesive, and film properties. For decades, starch has been industrially extracted from plant-based sources on a large scale and processed for a wide range of industry sectors, such as food, cosmetics, paper, textiles, and pharmaceuticals.^15^ Furthermore, the renewable and biodegradable nature of starch makes it an attractive candidate as a green dispersing agent. In addition, graphene is an excellent filler that can be used to strengthen the electrical, mechanical, and barrier properties of the starch. The combination of these two materials provides tremendous opportunities for various novel applications. In general, starch is composed of two polysaccharides known as amylose and amylopectin. The amylose is a linear polysaccharide of d-glucose units that are joined by repeating glycosidic α-1,4 links and can thereby self-associate into helical conformations with a hydrophobic core. In contrast, the amylopectin is a branched polysaccharide with double helical side chains that are joined to its backbones by glycosidic α-1,6 links. The ratio of amylose and amylopectin contributes to the semicrystalline nature of the starch granule and its properties.

In the last 20 years, starches from a wide range of botanical sources have been explored as dispersing agents for allotropes of carbon. For instance, the earliest work focused on the stabilization of single-walled carbon nanotubes (SWCNTs) by amylose^16^ and amylopectin^17^ in aqueous systems. The stabilization of the SWCNTs was assigned to hydrophobic interactions between the SWCNT surface and the helical core of the amylose, while that of the hydrophobic sites on the amylopectin. Furthermore, the SWCNTs were functionalized by different polar groups via acid treatments to increase their hydrophilicity and interfacial adhesion with starch.^18,19^ However, since the rise of graphene,^20^ the interest shifted toward graphene and its derivatives. In the pioneer work of Li et al. and Ma et al., an aqueous solution of graphene oxide (GO) was directly added to starch^21^ and starch/chitosan mixture,^22^ respectively. A distribution of oxygen groups on the GO surface increases its hydrophilicity and thereby eliminates the need for dispersing agents in aqueous systems. However, impurities on the surface substantially limit the properties of graphene. To restore these properties, starch was also explored as a reducing agent to remove these oxygen groups and functionalize the resulting reduced GO (RGO).^23,24^ In general, the interfacial interaction between GO/RGO and starch is commonly attributed to hydrogen bonding via their oxygen groups, respectively.^25−27^ While these oxygen-rich GO/RGOs resemble the geometric dimension of pristine graphene, their surface properties and functions are substantially different compared to the pristine graphene. Therefore, the same stabilization mechanism is not expected in the dispersion of graphene. To the best of our knowledge, the earliest work on graphene as a starting material reported starch-based films that showed improved properties at low graphene loading, while the opposite trend was observed at high loading as a result of graphene aggregation.^28,29^ In the last few years, the increasing availability of commercial graphene powders has sparked interest to explore graphene as a filler in starch-based formulations for various applications. However, the dispersion of graphene in the presence of starch is not well understood, in particular, the processing conditions and long-term stability.

In this paper, we report the dispersion of graphene in water using starch nanoparticles as a dispersing agent. These starch nanoparticles were prepared from unmodified starch granules via gelatinization and ultrasonication. To gain a better understanding of the stabilization mechanism, we used a starting graphene powder with a low oxygen content with close resemblance to that of pristine graphene and studied the effects of different processing conditions to obtain stable starch–graphene sheets. The findings demonstrate that starch can disperse graphene in water without oxygen-rich functional groups.

## 2 Results and Discussion

**Figure 1:**
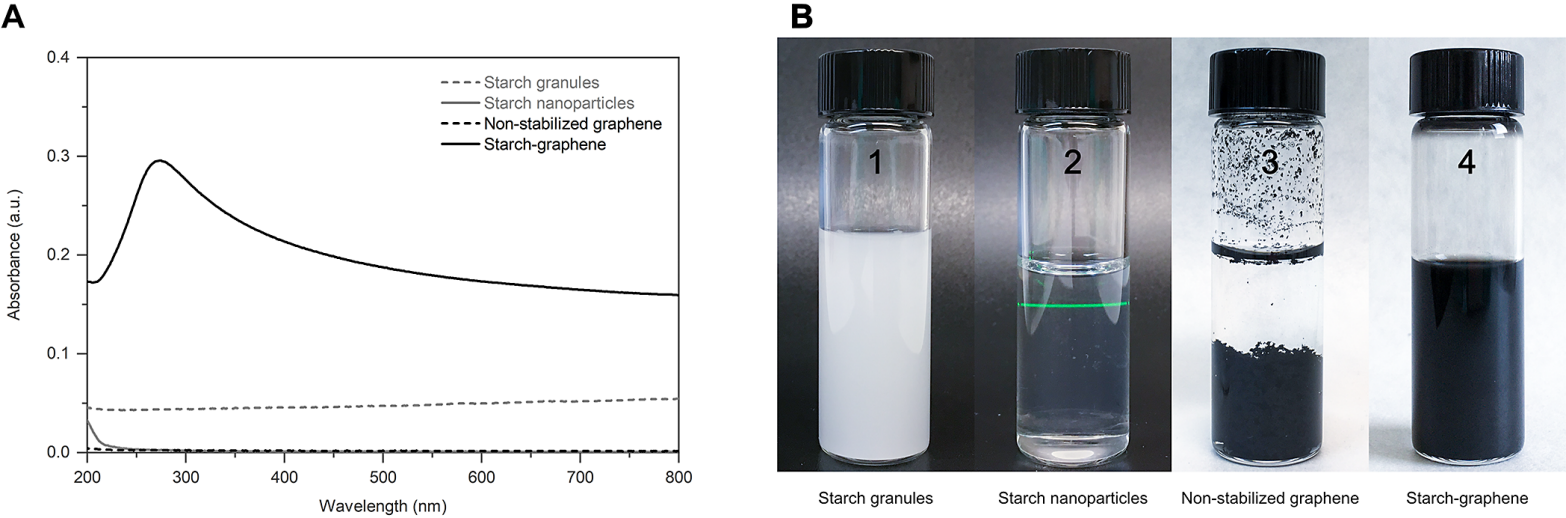
(A) UV–vis absorption spectra of starch granules (gray dashed), starch nanoparticles (gray solid), nonstabilized graphene reference (black dashed), and starch–graphene dispersion (black solid). (B) Photographs of a milky suspension of the starch granules (1), a more transparent solution of the starch nanoparticles demonstrating the Tyndall effect (2), a suspension of the nonstabilized graphene reference without starch (3), and the starch–graphene dispersion (4).

The relative amount of graphene that could be stabilized by starch nanoparticles was estimated via ultraviolet–visible (UV–vis) absorbance. UV–vis absorption spectra along with digital photographs of the starch–graphene dispersion are shown in Figure 1. As shown in Figure 1A, the absorbance baseline of the starch–graphene dispersion (black solid) increased from 0 to 0.17 au in the UV–vis range when graphene was stabilized by starch. Furthermore, a sharp absorption peak is observed at 273 nm, at which this peak position is attributed to π → π* transitions of conjugated aromatic rings, also commonly known as the fingerprint of dispersed graphene. In contrast, a nonstabilized graphene reference (black dashed) that was processed under the same conditions without starch gives a complete featureless absorption spectrum. As expected, the absence of starch leaves the graphene sheets to immediately restack in water, thus ruling out the stabilization of graphene via ultrasonication-induced defects that can be introduced after excessive ultrasonication.^30^ Similarly, starch references (gray dashed, solid) also give featureless absorption spectra, thus demonstrating that the features in the absorption spectra are exclusively originated from the dispersed graphene. As can be seen in the corresponding photographs in Figure 1B, the milky suspension of starch granules (photo 1) becomes more transparent after processing to starch nanoparticles (photo 2) and displays the Tyndall effect. Evidently, the nonstabilized graphene reference (photo 3) is clearly aggregated without starch, while the starch–graphene dispersion (photo 4) remains stable.

**Figure 2:**
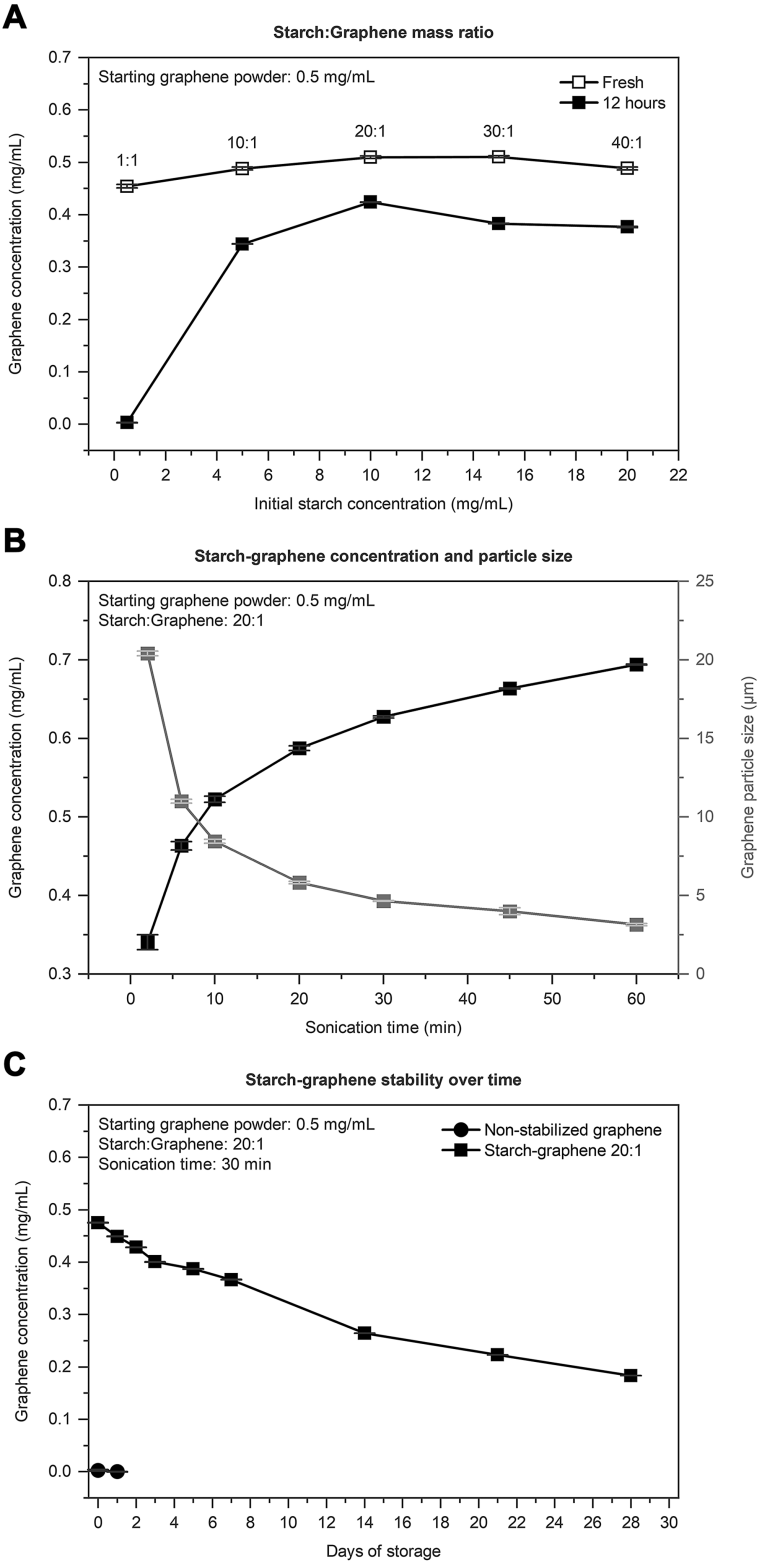
(A) Effect of initial starch concentration on the amount of dispersed graphene concentration while fresh (white squares) and after 12 h (black square). (B) Effect of sonication time on the dispersed graphene concentration (black square) and the graphene particle size (gray square). (C) Stability over time of the resulting starch–graphene dispersion (black square) compared to a nonstabilized graphene reference without starch (black circle).

The processing conditions of the starch–graphene dispersion were then systematically investigated in three steps, as summarized in Figure 2. First, the optimal mass ratio of starch/graphene (Figure 2A) was investigated by fixing the amount of starting graphene powder at 0.5 mg/mL in water and varying the starch concentrations between 0.5 and 20 mg/mL. The freshly prepared starch–graphene dispersions were equilibrated for 12 h under ambient conditions. After equilibration, the starch–graphene dispersions clearly showed that the dispersed graphene concentration increases with the starch concentration until a maximum is reached and then slightly drops with further addition of starch. The dispersion of the initial 0.5 mg/mL starting graphene powder was maximized at the starch concentration of 10 mg/mL (starch:graphene mass ratio of 20:1). Second, while keeping the optimal mass ratio fixed, the effect of sonication time on both the graphene concentration and the particle size was investigated (Figure 2B). The graphene concentration at the optimal starch/graphene mass ratio was further increased with sonication time. However, the particle size of the graphene sheets rapidly decreased with the sonication time until reaching 5 μm, below which the graphene sheets appeared less influenced by ultrasonication. An optimal sonication time of 30 min was chosen to obtain the highest graphene concentration while minimizing possible sonication-induced defects. Finally, the long-term stability of the optimized starch–graphene dispersion was evaluated over time by measuring the absorbance of a stationary sample that was stored at 4 °C (Figure 2C). The stability of the starch–graphene dispersion demonstrates a nonlinear behavior. Initially, we observe a decline in the graphene concentration for the first three days, after which the decline continues at a slower rate. According to Stokes’ law on the sedimentation rates of particles within a fluid medium, this trend can be attributed to the graphene sheets of larger particle size that sediment at a greater rate than those of smaller particle size by gravitational force. Overall, the starch–graphene dispersion remained stable for up to 1 month and showed no signs of phase separation. Besides, the starch–graphene dispersions could readily be redispersed.

**Figure 3:**
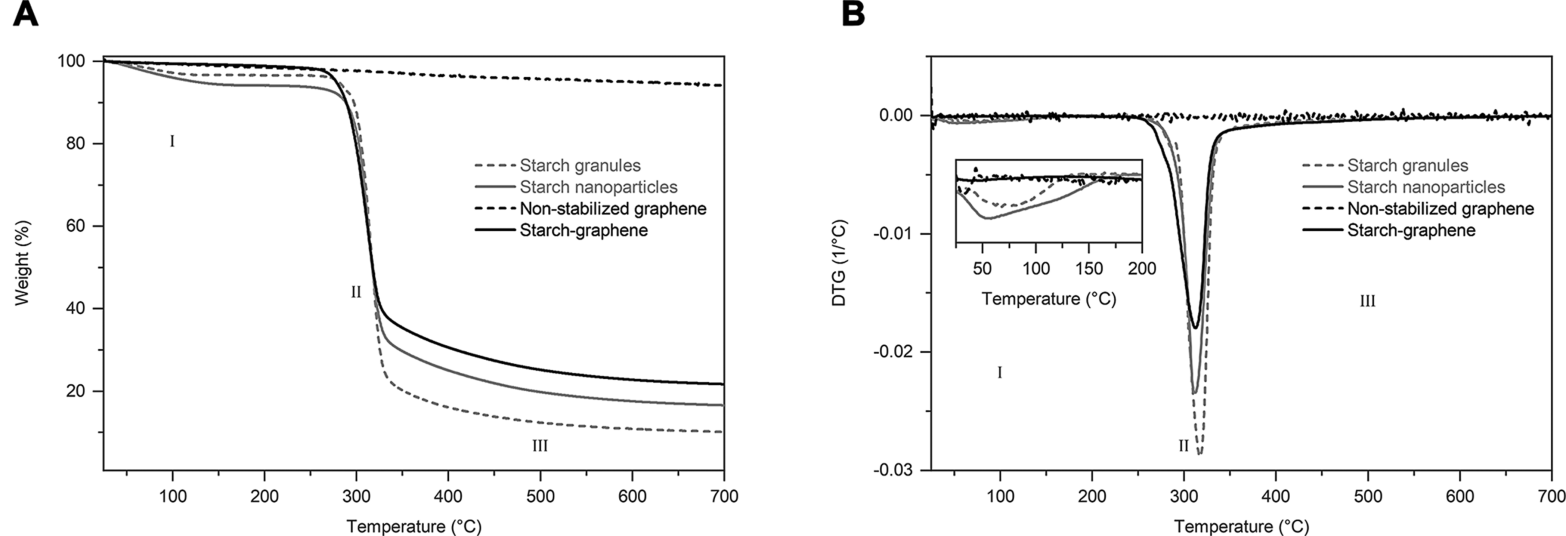
(A) TGA curves of starch granules (gray dashed), starch nanoparticles (gray solid), a nonstabilized graphene reference (black dashed), and starch–graphene sheets (black solid), alongside their corresponding (B) DTG curves. Inset: magnified view of the DTG curves between 25 and 200 °C, thus highlighting the dehydration steps.

To find the graphene concentration via optical absorbance, the attenuation coefficient for graphene is needed. For graphene, this coefficient was experimentally determined by constructing a calibration curve (S1) of the graphene absorbance against the graphene concentration. The total graphene concentration in this dispersion was then determined by thermal analysis (Figure 3). As shown in Figure 3A, the thermogravimetric analysis (TGA) curve of the graphene powder (black dashed) is relatively flat under a nitrogen atmosphere, thus indicating high degree of carbon purity.^31,32^ The starch granules (gray dashed) and the starch nanoparticles (gray solid), in contrast, show three decomposition regions. First, 25–170 °C (I) is the weight loss associated with the dehydration of weakly adsorbed water. Second, 200–400 °C (II) is the depolymerization of the starch backbone and accounts for the largest weight loss. Finally, third, 400 °C (III) and higher is the formation of carbonaceous residues, such as aromatic, aliphatic, and aldehyde groups.^33−38^ Among these carbonaceous residues, the aromatic groups are the dominant residuals after 600 °C.^39^ Therefore, we estimated the graphene content based on the residual weight difference between the starch–graphene and the starch nanoparticles in the flat region of the derivative thermogravimetric (DTG) curves at 700 °C. This yields a graphene concentration of 0.48 mg/mL in the purified starch–graphene dispersion and an attenuation coefficient of 3384 m^–1^ mg^–1^ mL.

In addition, TGA can also offer insight into the composition and thermal stability of the starch–graphene sheets. In the corresponding DTG curves (Figure 3B), we notice changes in the starch decomposition rates by the interaction with the graphene sheets in two regions. First, in the DTG region 25–170 °C (I), both the starch nanoparticles (gray solid) and the starch granules (gray dashed) show a broad peak, while the peak of the starch–graphene (black solid) is completely flat. In this region, the initial weight loss of both the starch references is between 3% and 6%, while that of the starch–graphene is substantially lower at 0.92%. The low weight loss indicates that graphene prevents weakly adsorbed water molecules onto the polysaccharides in the starch nanoparticles that otherwise can interact with water molecules via hydrogen bonding. Second, in the DTG region 200–400 °C (II), the temperature at which the rate of weight loss is maximum (*T*_max_) was lower for both the starch–graphene sheets (312 °C) and the starch nanoparticles (312 °C) compared to the starch granules (318 °C). In general, the shift to lower *T*_max_ is attributed to the higher surface area in the starch nanoparticles compared to the native starch granules, thus making the nanoparticles more susceptible to thermal decomposition. In the thermal studies by Aggarwal et al., starch granules revealed lower *T*_max_ after hydrolyzation to more porous granules with a higher surface area.^40^ The higher surface area was also responsible for lower *T*_max_ among different starch nanoparticles compared to their native granules. Qin et al. attributed the higher surface area to reduced particle sizes and disordered polysaccharide structures.^41^ At the molecular level, the linear polysaccharide amylose is known to decompose at lower temperatures than the branched amylopectin due to lower molecular weight and structural differences.^42,43^ As demonstrated, the maximum peak at *T*_max_ can provide insights into the starch surface characteristics and composition. In our work, the maximum peak of the starch–graphene is broader and asymmetric, which indicates that the decomposition of the starch nanoparticles occurs over a wider temperature range and is less volatile. This asymmetry is not observed in peak of the pure starch nanoparticles despite the same processing conditions. The processing of ultrasonication is known to cleave the polysaccharide chains, in particular, debranch the amylopectin, thus increasing the relative content of free amylose. However, both these polysaccharides can self-associate into more ordered structures and gradually larger networks, thus increasing the dissociation temperature. Therefore, the different thermal behavior of starch nanoparticles on the graphene surface implies that the starch–graphene interaction may sterically inhibit the free polysaccharides to self-associate into otherwise preferred polymorphic structures.

**Figure 4:**
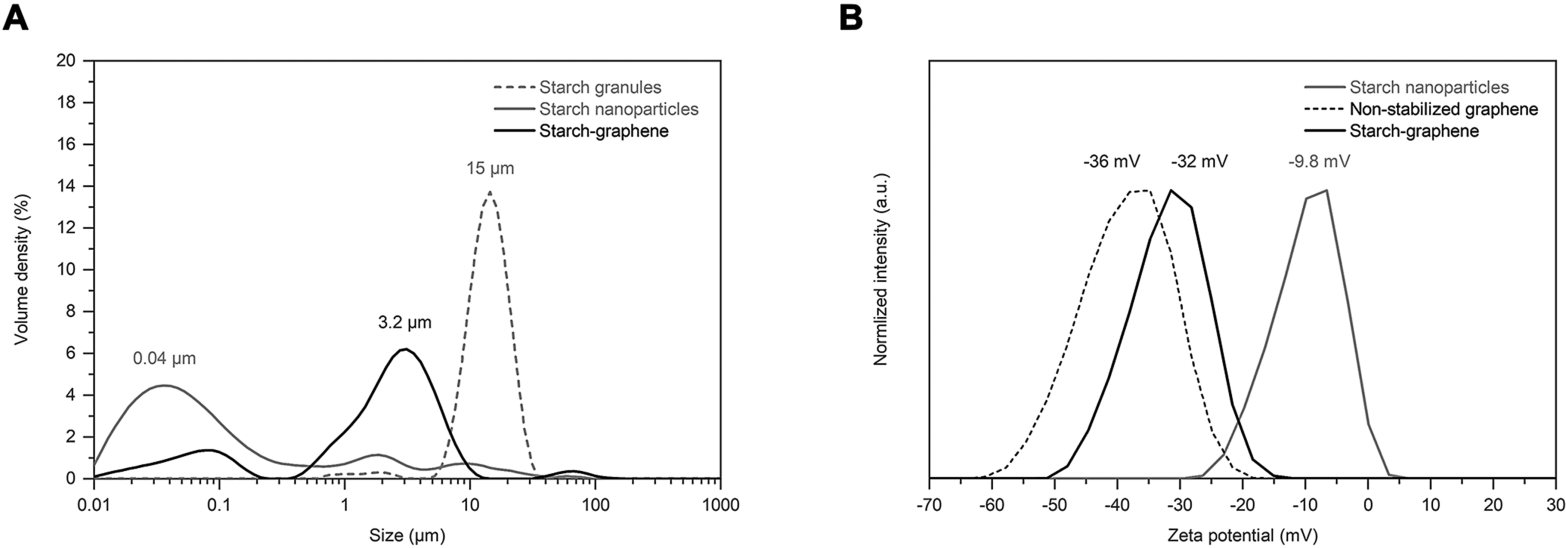
(A) Particle size distribution of starch granules (gray dashed), starch nanoparticles (gray solid), and starch–graphene sheets (black solid). (B) Zeta potential of the starch nanoparticles (gray solid), a nonstabilized graphene reference without starch (black dashed), and the starch–graphene sheets (black solid).

The particle size distribution of the starch–graphene sheets and the starch references were studied by a laser diffraction method (LDM), as shown in Figure 4A. The size distribution of the starting starch granules is sharp, with a maximum peak at 15 μm, which is of a similar size to that observed in other reports using LDM.^44,45^ The starch nanoparticles show a reduced the maximum peak to 40 nm, with a broader peak shape in the nanometer range. Furthermore, the starch nanoparticles also show weaker peaks in the larger particle size range. These weaker peaks could originate from various polymorphs of self-associated and cocrystallized starch polysaccharides. However, the starch–graphene sheets have a sharper size distribution with a maximum peak at 3.2 μm, as expected after 30 min ultrasonication.

To understand the stability of the starch–graphene sheets, the zeta potential of the starch–graphene dispersion is compared to that of the control samples that were prepared and measured under the same conditions (Figure 4B). The starch nanoparticles (gray solid) showed a negative zeta potential of −9.8 mV, thus revealing a weak net negative surface charge. A net negative surface charge in the same range has been reported before and was attributed to oxygen-containing functional groups on the polysaccharides.^46−49^ Interestingly, the nonstabilized graphene reference (black dashed) showed a negative zeta potential of −36 mV, while that of the starch–graphene sheets (black solid) was slightly increased to −32 mV. As demonstrated, the adsorption of starch nanoparticles reduced the net negative surface charge of graphene slightly. To the best of our knowledge, the zeta potential of starch-stabilized graphene materials of such low oxygen contents (O < 1.5%) has not been extensively investigated. Up to this point, Zhu et al. first reported starch-stabilized RGO with a negative zeta potential that gradually increased from a minimum of −27.8 up to −15.6 mV with a higher starch loading, in which the latter zeta potential value was close to that of the pure starch.^50^ Furthermore, Narayanan et al. also reported starch-stabilized RGO with a negative zeta potential that increased from −30.5 up to −21.5 mV with a higher starch loading.^51^ As reported by the authors, the higher starch loading gradually reduced the surface charge of RGO and consequently weakened the electrostatic repulsion between the sheets, thus resulting in aggregation. In our work, the graphene reference was easily aggregated despite its high magnitude of the zeta potential at −36 mV, while the starch–graphene sheets of lower magnitude of −32 mV remained stable. This implies that the stabilization of graphene was mainly attributed to the adsorbed starch nanoparticles rather than the surface charge, from which the magnitude of the zeta potential alone proved insufficient. The understanding of the surface charges on the RGO and other graphene derivatives is established, in which the negative charges are primarily attributed to different ionizable oxygen groups present on the sheets.^52^ However, the origin of the negative charges on the graphene sheets is still an ongoing debate. Several authors have also reported graphene in water with a negative zeta potential within the range of −45 and −30 mV at neutral pH.^53−56^ The authors have explained the origin to possible oxygen groups introduced at the graphene edges during ultrasonication^53,54^ and asymmetric adsorption of water ions on hydrophobic surfaces.^55,57^ Overall, we demonstrate that the adsorbed starch nanoparticles play a more important role in the stabilization of graphene than the net negative surface charge.

**Figure 5:**
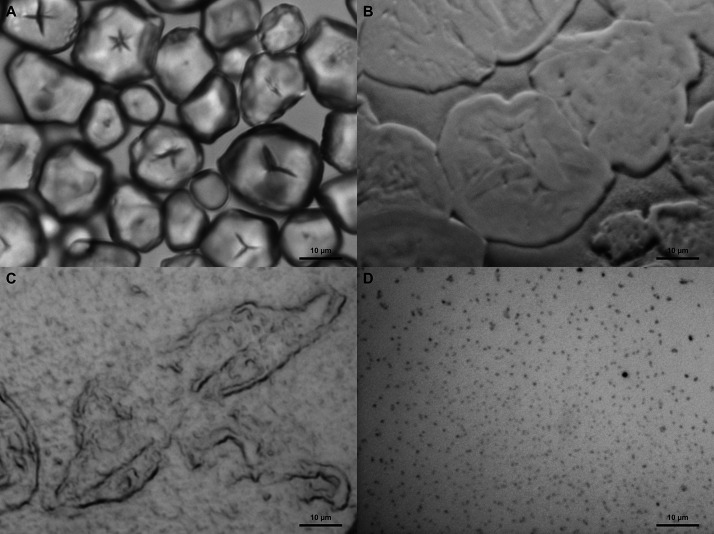
Optical microscopy images of (A) native corn starch granules in the size range of 15 μm, (B) gelatinized starch granules after 95 °C for 20 min, (C) ruptured starch granules after 3 min ultrasonication, and (D) clusters of the resulting starch nanoparticles after additional 30 min ultrasonication.

The preparation of the starch nanoparticles via ultrasonication was systematically studied on glass substrates by optical microscopy, as displayed in Figure 5. First, starch granules (Figure 5A) in an aqueous suspension initially showed polyhedral shapes in the size range of ∼15 μm and then swollen round shapes after boiling. The swollen granules (Figure 5B) were then further ruptured via ultrasonication until the suspension changed from white turbid to more clear solution. However, partly ruptured granular structures that remain after the ultrasonication, commonly known as granule “ghosts” (Figure 5C), were removed through sedimentation. From the final starch solution (Figure 5D), round-shaped particles in the size range of 100 nm can be observed after drying in room temperature. These round-shaped particles resemble the clusters of starch nanoparticles that are commonly prepared via ultrasonication.^58^ A similar morphology of starch nanoparticles has also been observed by means of other preparation methods, such as chemical hydrolysis,^59^ enzymatic hydrolysis,^60^ and precipitation.^61^

**Figure 6:**
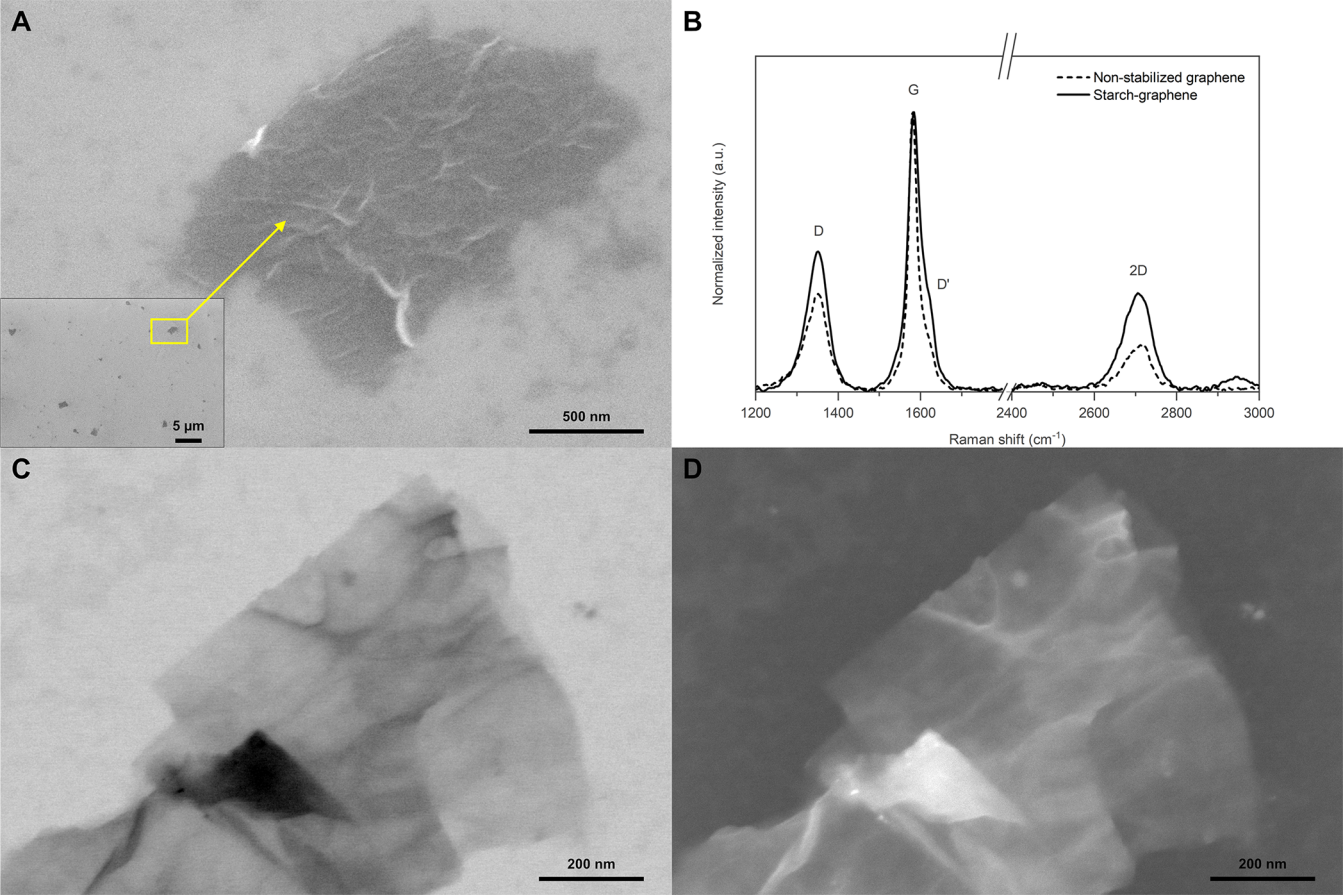
(A) SEM image of a representative starch–graphene sheet, (B) Raman spectra of the starch–graphene sheets (black solid) compared to a nonstabilized graphene reference (black dashed), and STEM images of a starch–graphene sheet in (C) BF and (D) HAADF, respectively.

The morphology of the starch–graphene sheets was then studied by high-resolution imaging using SEM, as shown in Figure 6. In contrast to the morphology of the granule ghosts, the starch–graphene sheets are clearly thinner and have well-defined edges. The lateral size of the starch–graphene sheets is in the range of 3 μm, which is consistent with the particle size measurement by the LDM. A representative starch–graphene sheet at higher magnification (Figure 6A) also reveals various surface features, such as wrinkles and self-folding events.

The structural changes in the starch–graphene sheets by the stabilization of starch nanoparticles were studied by Raman spectroscopy. For this purpose, the Raman spectra of starch–graphene are compared to a nonstabilized graphene reference that was prepared without starch under the same conditions (Figure 6B). Under such conditions, the most prominent peaks of the graphene reference were located near 1350 cm^–1^ (D peak), 1580 cm^–1^ (G peak), and 2700 cm^–1^ (2D peak). Among these peaks, the D peak is related to the breathing vibrations of aromatic carbon rings and becomes active by disorders, such as grain boundaries, vacancies, or sp^3^-related defects. The G peak from the in-plane stretching of carbon atoms in the ring is a common feature in all sp^2^-hybridized carbon systems.^62^ Therefore, the degree of structural disorders in the graphene sheets can be estimated by the intensity ratio *I*(D)/*I*(G) of these two peaks. In our work, the *I*(D)/*I*(G) of the graphene reference is 0.4 and that of the starch–graphene sheets increased to 0.6 after stabilization by starch nanoparticles, thus indicating an increased degree of disorders. Furthermore, the nature of these disorders can be probed by the intensity ratio of the D peak over a weak shoulder peak around 1620 cm^–1^ (D′ peak). These two peaks are both disorder-related peaks from the intervalley scattering and intravalley scattering, respectively.^62^ The values of the intensity ratio *I*(D)/*I*(D′) from these two peaks can be used to describe different types of disorders that are distinguished at 3.5 (boundaries), 7 (vacancies), and 13 (sp^3^ defects).^63^ In our work, the height of the D′ peak was read from a deconvoluted peak shape using the Voigt function. As a result, the intensity ratio *I*(D)/*I*(D′) of the starch–graphene and the graphene reference is 2.4 and 2.6, respectively. The relatively low intensity ratio implies that the disorders on the graphene sheets mostly originate from boundary effects, such as various surface features and edges. The density of such disorders can increase during ultrasonication, in particular, the density of edges as a result of reduced particle size. Moreover, the effects of disorders are known to change the peak positions and shape of both the G peak and the 2D peak.^64,65^ Therefore, the use of these two peaks to estimate the number of graphene layers requires caution. Gupta et al. and Wang et al. reported a shift toward the higher frequencies up to 1587 cm^–1^ for a defect-free monolayer graphene sheet.^66,67^ According to this concept, the peak position of the starch–graphene sheets at 1584 cm^–1^ reflects bilayer graphene. Alternatively, the 2D peak is the overtone of the D peak and does not require any disorders to be activated. In this concept, the number of graphene layers can instead be estimated based on the full width at half-maximum (FWHM).^68^ For the starch–graphene sheets, the 2D peak was fitted into a Lorentzian peak with an FWHM of 66 cm^–1^, thus reflecting five-layer graphene. As demonstrated, the estimation can vary as a result of various types of disorders. Overall, the Raman spectra indicate that starch–graphene sheets are within the range of few-layered graphene sheets with a relatively low amount of disorders from boundaries.

The morphology of the starch–graphene sheets was further studied in STEM using both the bright-field (BF) and the HAADF detectors, respectively. In the BF image (Figure 6C), a representative starch–graphene sheet shows transparent graphene layers and contrast difference between the self-folding effects. Furthermore, the contrast difference also illuminates few round-shaped nanoparticles with a size of tens of nanometers on the graphene surface. Similarly, the illumination of these nanoparticles in the HAADF image (Figure 6D) indicates that the starch nanoparticles have a different crystal structure and mass than the underlying graphene layers.

**Figure 7:**
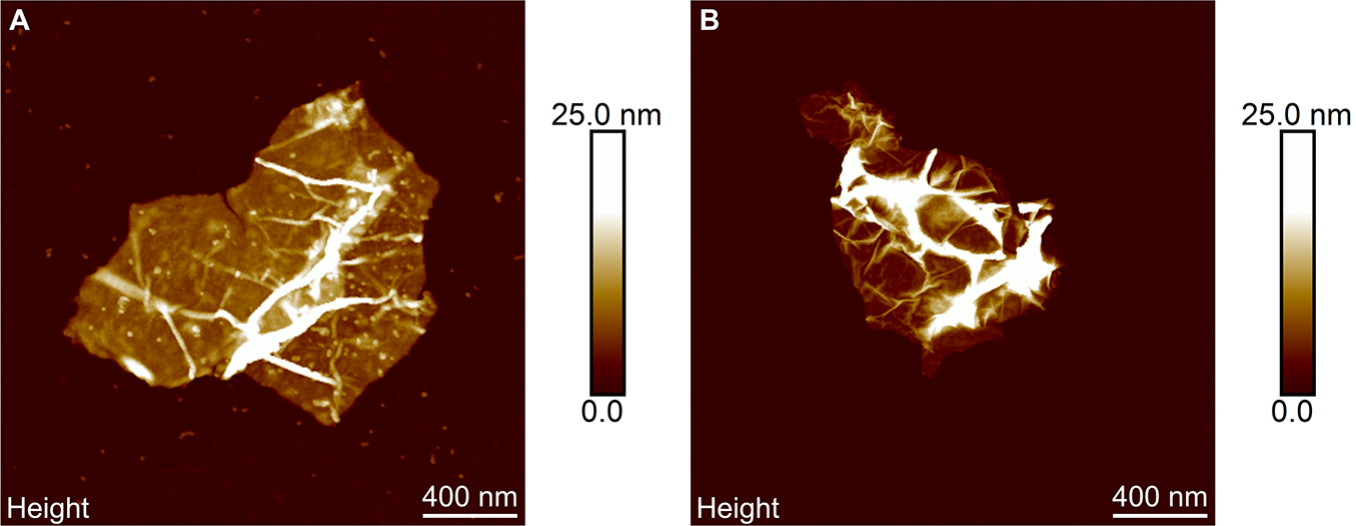
AFM topographical images of (A) a starch–graphene sheet and (B) a nonstabilized graphene reference without starch.

The starch nanoparticles on the graphene surface were further studied at the nanoscale using AFM. The topographical image of a starch–graphene sheet (Figure 7A) is compared to that of a nonstabilized graphene reference (Figure 7B). The image of the graphene reference without starch displays a clean surface with dense surface features, such as ripples, wrinkles, and crumples. In contrast, the starch–graphene sheet clearly displays round-shaped starch nanoparticles that are spread on the graphene surface. In addition, a few of these starch nanoparticles can also be observed in the surrounding on the mica substrate. The adsorbed starch nanoparticles on the graphene surface have a uniform size distribution around 5 nm in height, which creates a distance between two graphene sheets that can prevent van der Waals attractive forces. Therefore, the starch nanoparticles may act as an energy barrier to prevent the restacking of adjacent graphene sheets. Moreover, to confirm that these nanoparticles originate from starch, we also studied the topography of a starch reference that was prepared under the same conditions as the starch–graphene dispersion (S2). As expected, the topographical image of the pure starch reference displays similar round-shaped starch nanoparticles in the same size range as the observed nanoparticles on the graphene surface. Evidently, we confirm the adsorption of starch nanoparticles on the graphene surface.

**Figure 8:**
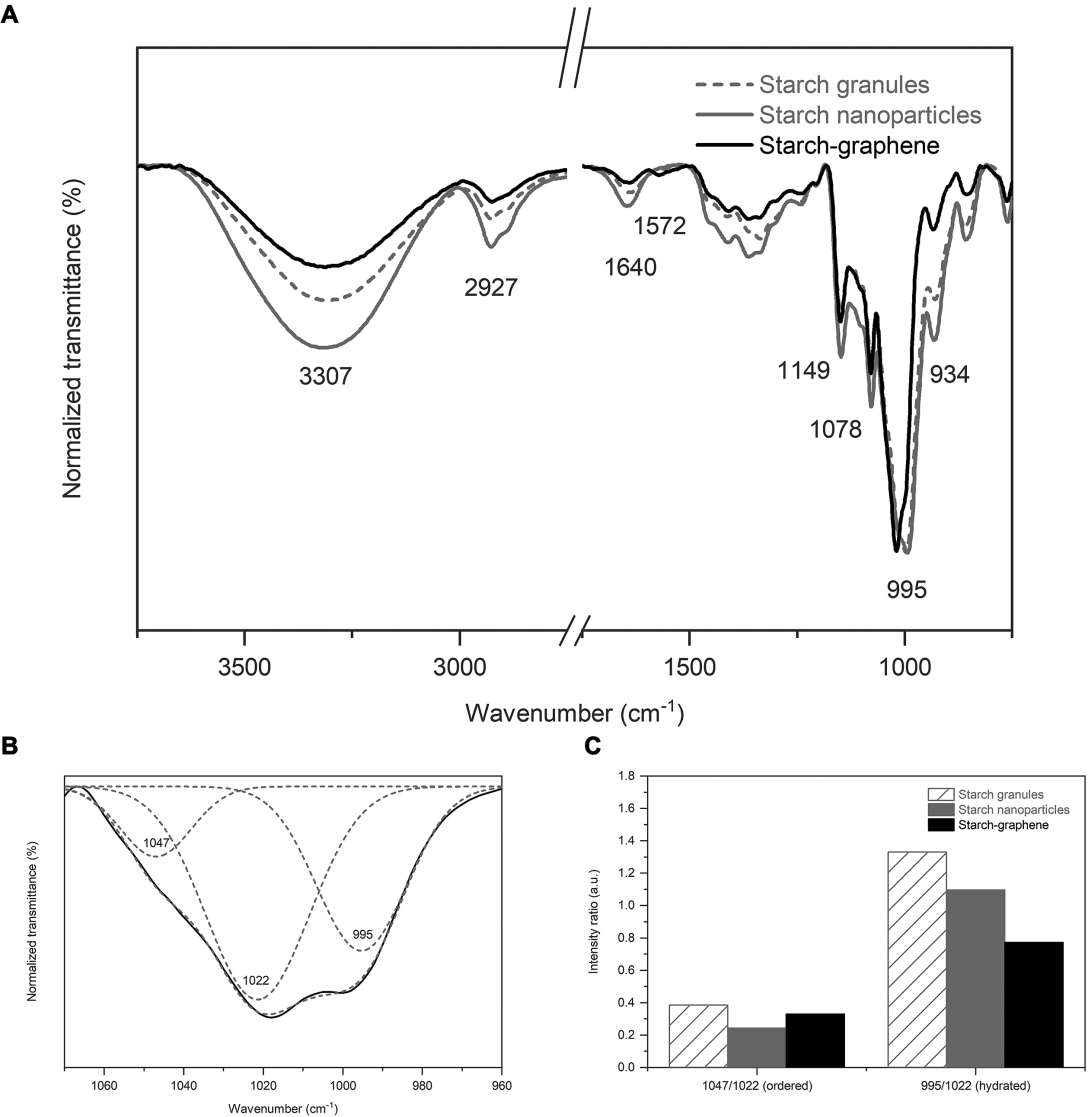
(A) FTIR spectra of starch granules (gray dashed), starch nanoparticles (gray solid), and starch–graphene sheets (black solid). (B) An example of deconvoluted peaks of the starch–graphene sheets in the starch fingerprint region. (C) Intensity ratios of 1047/1022 (ordered starch structures) and 995/1022 (hydrated starch structures).

The physiochemical interaction of the starch nanoparticles on the graphene surface was studied by FTIR. As shown in Figure 8, comparing the starch–graphene (black dashed) to the starch nanoparticles and the starch granules (gray dashed, solid), we observe two major differences. First, an additional peak emerged at 1572 cm^–1^ in the starch–graphene, which is not present in the spectra of the starch references. This additional peak is tentatively attributed to C=C stretching vibrations in conjugated aromatic rings, thus indicating the presence of graphene.^51^ Second, the presence of graphene also changed the vibrational modes of starch. The peaks of starch are typically identified at 1149 cm^–1^ (C—O, C—C stretching, and partially C—O—H contributions), 1078 cm^–1^, and 995 cm^–1^ (C—O—H bending).^69^ Among these peaks, the maximum peak at 995 cm^–1^ was substantially narrowed and shifted to the higher wavenumber of 1019 cm^–1^ by graphene. To investigate this peak further, we performed deconvolution and observed three overlapping peaks centered around 1047, 1022, and 995 cm^–1^, as shown in Figure 8B. These peaks are known to provide insight into the relative degree of starch structures. For instance, the peak at 1047 cm^–1^ is sensitive to the degree of ordered starch structures, whereas the peak at 1022 cm^–1^, in contrast, is associated with amorphous structures. Finally, the peak at 995 cm^–1^ is mainly associated with the intra- and intermolecular hydrogen bonding of the hydroxyl group at C(6)—O—H, which makes this peak sensitive to the water content.^70^ Commonly, the intensity ratio of 1047/1022 and 995/1022 are indicators of the short-range crystalline and hydrated starch structures, respectively (Figure 8C). In our work, the crystalline-to-amorphous ratio of 1047/1022 is determined in the increasing order: starch nanoparticles (0.25), starch–graphene (0.33), and starch granules (0.37). As expected, this indicates that the structures of polysaccharides are more ordered within the starch granules than in the pure starch nanoparticles. Furthermore, the short-range crystallinity in the pure starch nanoparticles was easily disrupted by gelatinization and ultrasonication, while the same processing conditions were less effective against the starch nanoparticles on the graphene surface. Moreover, the strong starch–graphene interaction is reflected in the hydration behavior of starch. The hydration-to-amorphous ratio of 995/1022 is in the increasing order: starch–graphene (0.74), starch nanoparticles (1.10), and starch granules (1.31). As demonstrated, the degree of hydrated starch structures was substantially lower in the starch–graphene than in the starch references. At the molecular level, this implies that the starch–graphene interaction has a disruptive effect on the hydrogen bonding network of the starch nanoparticles in water. That is, the hydrophobic surface of graphene changed the molecular environment and space available for the hydroxyl groups of starch to engage in hydrogen bonding with nearby water molecules.^70,71^ As a result, the amount of water content is substantially reduced in the starch–graphene sheets, which is in agreement with the thermal decomposition observed in TGA.

Further evidence to support this observation can be found at the peak of 1640 cm^–1^, which reflects the vibrational modes of bound water in the starch. Comparing the intensity of this peak relative to that of the maximum peak (1640/1022), the amount of bound water is substantially lower in starch–graphene than that in the starch references, thus supporting the reduced ability of starch nanoparticles to retain water. In addition, a prominent peak at 3307 cm^–1^ is mainly attributed to the vibrational stretching of free, intramolecular, and intermolecular interactions of the hydroxyl groups in starch. Therefore, peak shift and broadening to the shorter wavenumbers have been used as an indicator to detect hydrogen bonding between graphene derivatives and starch. However, in our work, the peak position varied only slightly in the increasing order: starch granules (3307 cm^–1^), starch–graphene (3316 cm^–1^), and starch nanoparticles (3318 cm^–1^). Similarly, the peak shapes remained the same with an average FWHM of 363 ± 4 cm^–1^. As demonstrated, the interfacial interaction between the graphene and the starch is not evident for hydrogen bonding, thus pointing to other noncovalent interactions that are more thermodynamically favored.^72^

**Figure 9:**
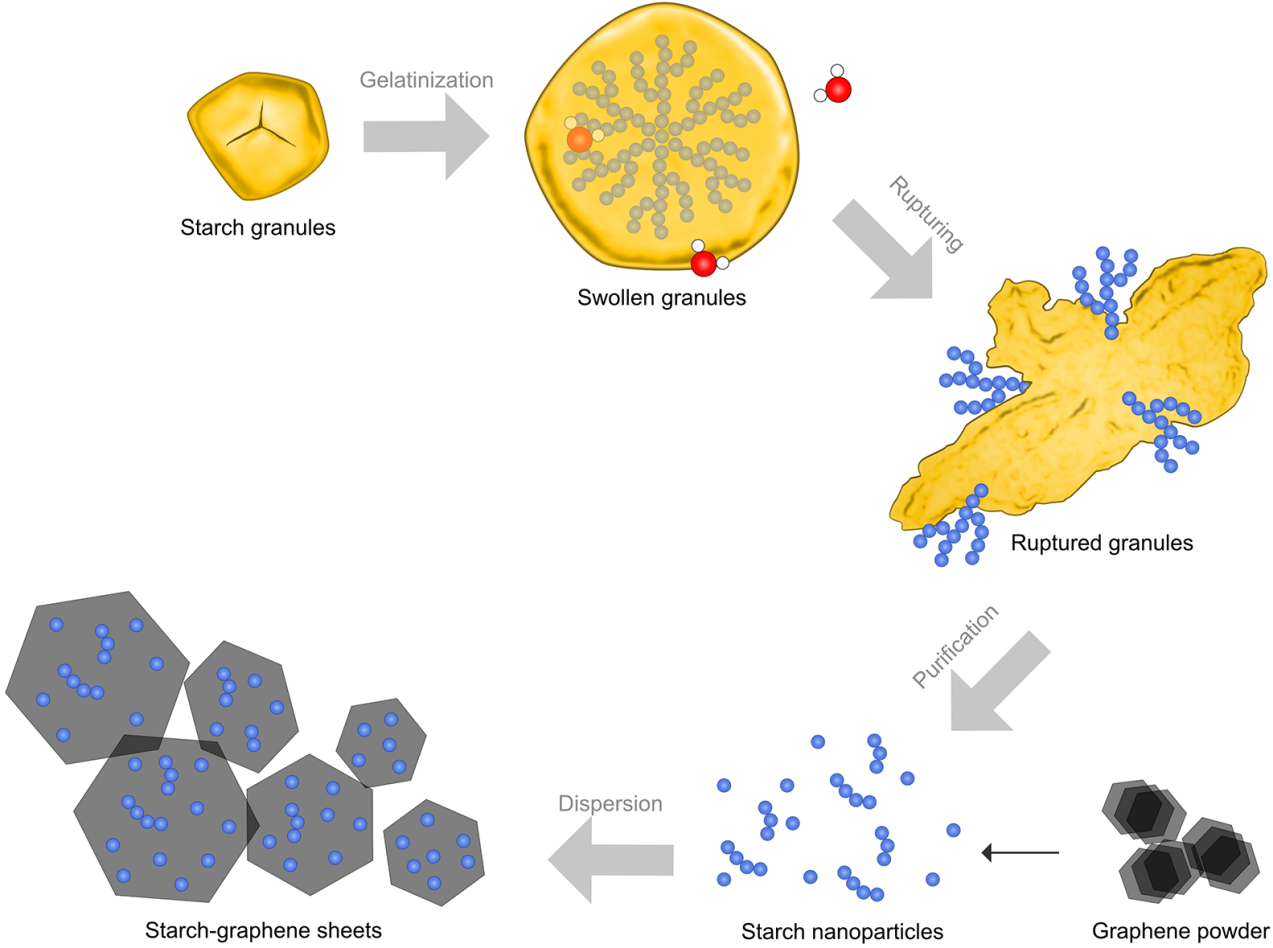
Illustration of the stabilization mechanism of the starch–graphene dispersion.

Based on the overall findings, we conjecture a mechanism for starch-stabilized graphene dispersions, as illustrated in Figure 9. In brief, the intra and intermolecular interactions between starch polysaccharides within the starch granules become disrupted during gelatinization and ultrasonication in water, whereby the starch granules begin to rupture and leach the starch polysaccharides. Naturally, these leached polysaccharides strongly interact with nearby water molecules (hydrated starch) via hydrogen bonding. However, in the presence of graphene, the graphene competitively interacts with water molecules for the starch polysaccharides in the stabilization process. These steps lead to the observed preferential adsorption of the starch nanoparticles on the graphene surface, thus providing mainly steric stabilization.

## 3 Conclusions

Aqueous graphene dispersions were prepared using starch nanoparticles as a dispersing agent. The effectiveness of these starch nanoparticles was mainly dependent on the initial starch concentration and sonication time. The optimal concentration between starch and graphene was identified at 20 times more starch than graphene and the sonication time of 30 min. The obtained starch–graphene sheets were mainly few-layer graphene in the size range of 3.2 μm with a relatively low amount of defects. At the nanoscale, the topographical images of the starch–graphene sheets confirmed that starch nanoparticles decorated on the graphene surface with a height around 5 nm. These starch nanoparticles on the graphene surface provided steric repulsion against the attractive van der Waals forces acting on adjacent graphene sheets and consequently extended the dispersion stability for up to 1 month. Moreover, the starch–graphene interaction revealed that the graphene inhibited the ability of starch to retain water. The reduced starch–water interaction was reflected in the substantial changes to the C—O—H vibrational modes of starch at 995 cm^–1^. These findings conclude that graphene without oxygen-rich functional groups can be dispersed in water, thus providing more insights into the preparation of environmental-friendly aqueous graphene dispersions. The combination of these two excellent materials, graphene and starch, in the aqueous dispersion provides tremendous opportunities for packaging applications.

## 4 Experimental Methods

### 4.1 Materials and Reagents

Graphene powders were purchased from Avanzare Innovacion Tecnologica, S.L. (Logroño, Spain). This graphene grade is an RGO with a low oxygen content (<1.5%), as provided by the supplier. Unmodified corn starch powder (S4126) containing 27% amylose and 73% amylopectin was purchased from Sigma-Aldrich. Milli-Q water was used for all the aqueous dispersions.

### 4.2 Preparation of Starch Nanoparticles and Starch–Graphene Dispersions

The starch nanoparticles were prepared from native corn starch via gelatinization and ultrasonication. In general, all aqueous suspensions of starch powder in the concentration range of 0.5–20 mg/mL were heated at 95 °C for 20 min under constant stirring at 1000 rpm. After the gelatinization step, the suspensions were treated by ultrasonication (Sonics Vibra-Cell VCX 750, probe tip diameter: 6 mm, frequency: 20 kHz, and amplitude: 40%) for 3 min and then cooled to room temperature. Subsequently, the suspensions were centrifuged at 1500 rpm for 5 min, whereby the supernatants containing the starch nanoparticles were extracted. After obtaining more clear starch solutions, a fixed amount of 5 mg starting graphene powder was added and then dispersed by ultrasonication. The optimal processing conditions of the starch–graphene dispersion were investigated by varying both the initial starch concentration (0.5, 5, 10, 15, and 20 mg/mL) and the ultrasonication time (2, 6, 10, 20, 30, 45, and 60 min). The final starch–graphene dispersion was purified from residual graphene aggregates by centrifugation at 1000 rpm for 10 min and then collecting the supernatant. The resulting graphene dispersion was used for all characterizations.

### 4.3 UV–Vis Spectroscopy

UV–vis absorption spectra were recorded in quartz cuvettes using a UV–vis spectrophotometer (PerkinElmer LAMBDA 650). Each sample was diluted by a factor of 100 and then equilibrated for 30 s prior to measurement. The concentration of graphene was determined from the optical absorbance, according to Beer–Lambert’s law *A* = α*lc*. In this expression, the absorbance (*A*) is proportional to the attenuation coefficient (α), light path length (*l*), and the concentration (*c*). The unknown attenuation coefficient, α, for starch–graphene dispersions was extracted by plotting the absorbance per light path length at the wavelength of 660 nm, (*A*_660_/*l*) against the graphene concentration, *c*, and then reading the slope from a fitted linear regression. For this purpose, an aliquot of the starch–graphene dispersion was serially diluted into six samples, from which the absorbance of each sample was measured. To find the graphene concentration in these dilutions, a known volume of the aliquot was first dried in an oven at 105 °C for 24 h. The remaining solid content from this aliquot was weighed after cooling to calculate the total concentration of both the starch and the graphene, in which the graphene part was estimated by TGA
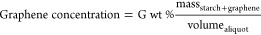


### 4.4 Thermal Properties of Starch–Graphene

TGA analysis was performed in 70 μL alumina crucibles using a thermogravimetric analyzer (TGA 2 STARe System, Mettler Toledo). The dried sample size varied between 0.5 and 1 mg for graphene and 2–6 mg for starch. Each sample was equilibrated in an oven at 105 °C for 24 h prior to measurements. The measurements were recorded under a nitrogen atmosphere at a constant heating rate of 10 °C/min.

### 4.5 Particle Size Distribution

Particle size analysis was performed using a laser-diffraction-based particle size analyzer with a Hydro SV measurement cuvette (Mastersizer 3000, Malvern). Each wet sample was added to the cuvette under constant stirring at 500 rpm until the obscuration level reached 5–10% for the starch–graphene and starch granules, while below 5% for the starch nanoparticles. For each sample, a total of five measurements were recorded and then averaged. The volume-weighed particle size distributions are calculated by the software using Mie theory. In the calculations, different optical properties were used for the graphene (refractive index of 2.73 and absorption of 1.36) and the starch (refractive index of 1.53, absorption of 0.01).

### 4.6 Zeta Potential

The surface charge of the starch–graphene sheets was determined using a Zetasizer (Nano ZS, Malvern). The starch–graphene dispersion was diluted by a factor of 100 (∼0.01 mg/mL) and then equilibrated in a folded capillary cell for 30 s prior to measurements. A total of six measurements were recorded with a minimum of 20 runs and then averaged. Each sample measured a pH of 6.8 using a standard lab bench pH meter.

### 4.7 Structure and Morphology

The optical images of the starch morphology were captured on glass slides using a reflected light microscope (Optiphot-100s, Nikon) equipped with a Nikon 50× microscope objective. High-resolution images of the starch–graphene morphology were captured using an SEM/STEM (Quanta FEG 250, FEI). For the SEM samples, the starch–graphene dispersion was diluted by a factor of 100 and then spin-coated on cleaned Si/SiO_2_ substrates. The substrates were cleaned by ultrasonication in ethanol for 5 min followed by rinsing with water and finally oxygen plasma treatment for 1 min to increase the surface wettability. For the STEM sample, the same starch–graphene dilution was drop-cast onto a 200-mesh copper grid and then dried under ambient conditions overnight prior to imaging. All images were captured in both BF and dark field (DF) by the STEM detector at 10 kV.

### 4.8 Confocal Raman Microscopy

Raman analysis of the starch–graphene sheets was performed using a WITec alpha 300 system (WITec GmbH, Germany). The starch–graphene sheets and the nonstabilized graphene reference were prepared via spin-coating on cleaned Si/SiO_2_ substrates under the same conditions as the SEM samples. For each sample, the Raman spectra were recorded using the excitation wavelength of 532 nm and a low laser power of 2.5 mW. The typical integration time was in the range of 2 s. The acquired spectra of an area were then corrected for cosmic rays and then averaged in the WITec Project 5.1 software (WITec GmbH, Ulm, Germany). Finally, the baseline corrections were performed using the Peak Analyzer in the OriginPro 2020 software.

### 4.9 Atomic Force Microscopy

Topographical imaging of the starch–graphene sheets was performed in the PeakForce tapping mode using a MultiMode 8 (Nanoscope V controller) atomic force microscope (Bruker, Santa Barbara, CA). The surface of the starch–graphene sheets was scanned using a cantilever ScanAsyst-Air (Bruker) with a nominal spring constant of 0.4 N/m and a nominal tip radius of 2 nm. For comparison with the starch–graphene sheets, the control samples of both a starch reference and a nonstabilized graphene reference were prepared under the same conditions. The graphene reference without starch was prepared in an aqueous solution of 70 vol % ethanol to obtain partially suspended graphene. For each sample, 25 μL of a dilution (by a factor of 100) was drop-cast on a freshly cleaved mica surface and then spin-coated at 3500 rpm for 1 min.

### 4.10 Physiochemical Characteristics of Starch–Graphene

Spectroscopic analysis of the chemical interactions between graphene and starch was performed using an FTIR spectrometer in the ATR mode (Spectrum One, PerkinElmer). Dried samples were first equilibrated in an oven at 105 °C for 24 h prior to measurements. For each sample, a total of 16 scans were collected at a resolution of 4 cm**^–1^** and then averaged. The average spectra were baseline corrected and then deconvoluted by Gaussian fit in the OriginPro 2020 software.

## Supporting Information Available

UV–vis calibration curve of starch–graphene dispersions and AFM images of pure starch nanoparticles (PDF)
