# Supplementary material for: Surfactant-Free Stabilization of Aqueous Graphene Dispersions Using Starch as a Dispersing Agent
Source: ACS Omega. 2021 Apr 28;6(18):12050–62. doi: 10.1021/acsomega.1c00699 (PMC8154146; doi:10.1021/acsomega.1c00699)
Supplement: Supplementary file 1 — ao1c00699_si_001.pdf [file ao1c00699_si_001.pdf]

Supporting information for

Surfactant-Free Stabilization of Aqueous Graphene Dispersions Using Starch as Dispersing Agent

Wei Zhao[1,3], Abhilash Sugunan[1], Thomas Gillgren[2], Johan A. Larsson[2], Zhi-Bin Zhang[3], Shi-Li Zhang[3], Niklas Nordgren[1], Jens Sommertune[1], Anwar Ahniyaz[1]*

1 RISE Research Institutes of Sweden, SE-114 86 Stockholm, Sweden.

2 BillerudKorsnäs AB, SE-718 80 Frövi, Sweden.

3 Division of Solid State Electronics, Department of Electrical Engineering, Uppsala University, SE-751 03 Uppsala, Sweden

S1. Attenuation coefficient of starch-graphene sheets

The graphene concentration in the starch-graphene dispersion after purification was estimated via its optical absorbance, according to Beer–Lambert's law A = αlc. The unknown attenuation coefficient, α, for starch-graphene dispersion was experimentally determined by plotting the absorbance per light path length (A_{660}/l) of six serially diluted dispersions against their graphene concentration, c, and then reading the slope from a fitted linear regression, as shown in Figure S1. The attenuation coefficient for graphene was determined to 3384 mL mg^{-1} m^{-1}.

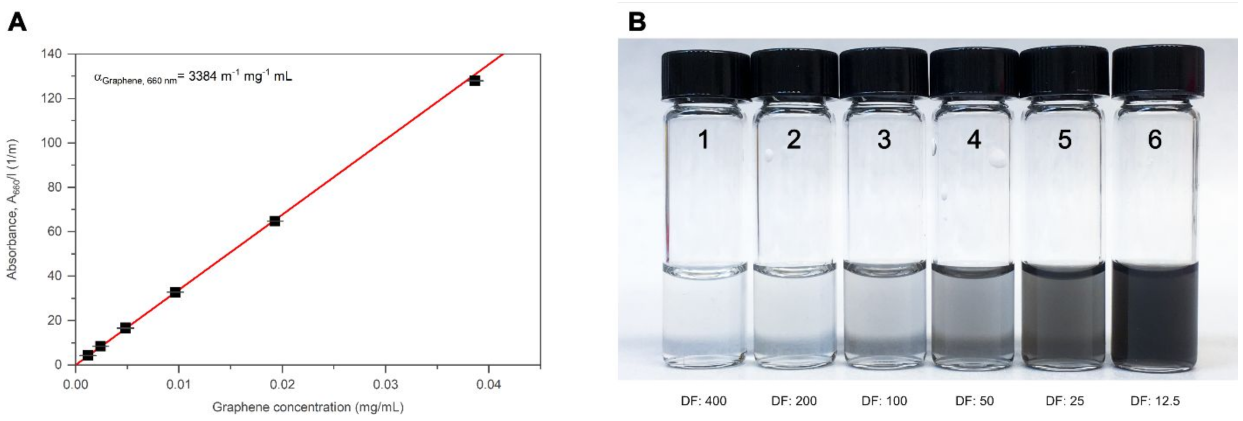

Figure S1. A) A calibration curve of serially diluted starch-graphene dispersions and their absorbances plotted against the graphene concentration. B) A photograph of the serially diluted starch-graphene dispersions 1-6 as read from left to right, showing dilution factor (DF) 400, 200, 100, 50, 25, and 12.5, respectively.

S2. AFM of starch nanoparticles

Topographical images of starch nanoparticles prepared under the same conditions as the starch-graphene dispersions (Figure S2). These starch nanoparticles were spin-

coated on a mica substrate and show a height around 5 nm that is consistent with the starch nanoparticles adsorbed on the graphene sheets.

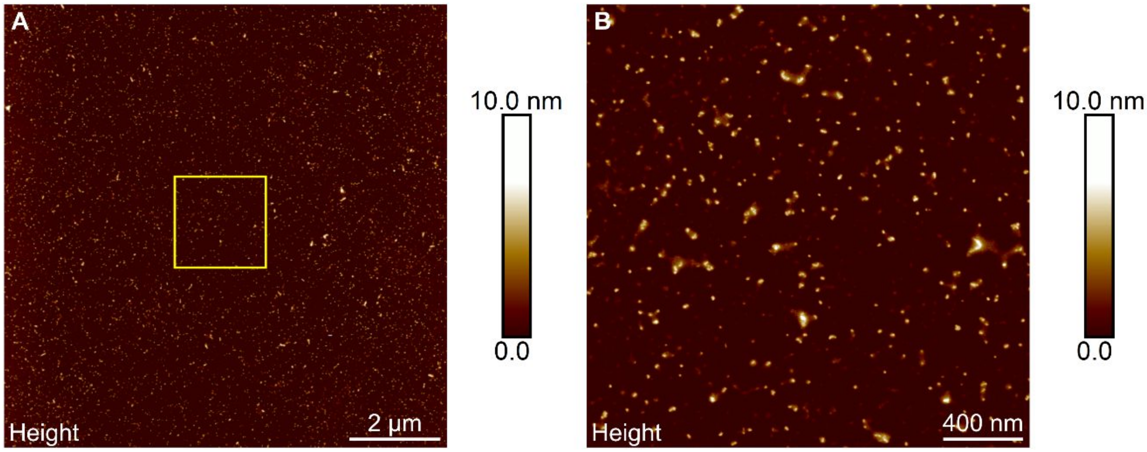

Figure S2. AFM topographical images of pure starch nanoparticles deposited on a mica substrate within an area of A) 10 μm x 10 μm and B) magnified 2 μm x 2 μm of the yellow box.
